# Acknowledgment to Reviewers of *Biosensors* in 2020

**DOI:** 10.3390/bios11020028

**Published:** 2021-01-20

**Authors:** Biosensors Editorial Office

**Affiliations:** MDPI AG, St. Alban-Anlage 66, 4052 Basel, Switzerland

Peer review is the driving force of journal development, and reviewers are gatekeepers who ensure that *Biosensors* maintains its standards for the high quality of its published papers. Thanks to the cooperation of our reviewers, in 2020, the median time to first decision was 14 days and the median time to publication was 35 days. The editors would like to express their sincere gratitude to the following reviewers for their precious time and dedication, regardless of whether the papers were finally published:
A. A. Munoz, RodrigoMacDonald, Ian MAdhikari, Bal RamMaior, IoanaAhn, Ji-YoungMakarian, KamranAlam, Arif UlMalla, SpundanaAlamaniotis, MiltiadisMałolepszy, ArturAlberto, NéliaManagò, StefanoALDEWACHI, HASANManderville, Richard A.Alfinito, EleonoraMao, HongjuAli, Md AzaharMarasso, Simone LuigiAmine, AzizMarchetti, MarialauraAminifar, AmirMartinelli, EugenioAnfossi, LauraMasarati, PierangeloAnishchenko, LesyaMatejec, VlastimilAntunes, Joana C.Matko, VojkoAphale, AshishMatowicka-Karna, JoannaAraujo, RosaMattoscio, DomenicoArino, ChristinaMechthild, Ladwig-WiegardAspholm, MarinaMeiyazhagan, Ashok KumarAvanaki, KamranMejía-Salazar, Jorge RicardoAwan, Shakil AhmedMendes, JoaquimAydemir, NihanMercader, JosepBadea Doni, MihaelaMercante, LuizaBagnato, Vanderlei SalvadorMerle, Géraldine E.Bala, CameliaMerrin, JackBalusamy, BrabuMiao, QingqingBaluta, SylwiaMicha, DimitraBañuelos, JorgeMicheli, LauraBarberis, AntonioMigliozzi, DanielBarbosa, Ana IsabelMilano, FrancescoBarek, JiriMin, Jun-HongBaron, Julianne L.Minas, GraçaBartsch, RonnyMironenko, AleksandrBashkatov, Alexey NikolaevichMishin, Alexander S.Bawage, SwapnilMishra, AvanishBeltsios, KonstantinosMishra, Geetesh K.Bertemes-Filho, PedroMitchell, John S.Bertocci, FrancescoMolognoni, DanieleBertoncello, PaoloMondal, SudipBessmertnykh-Lemeune, AllaMontes, RaquelBhatt, PriyankaMoon, Byeong-UiBi, LanrongMorales Narváez, EdenBian, ChaoMorel, Chantal M.Bianchi, VittorioMoretto, Ligia MariaBier, FrankMoro, GiuliaBikiaris, DimitriosMourzina, YuliaBishop, Greg W.Movafaghi, SanliBok, SanghoMu, YingBonardd, SebastianMukhopadhyay, SubhasBourillot, EricMuniz-Miranda, MaurizioBrad, RemusMuranaka, AtsuyaBraga, Guilherme S.Myasoedova, Tatiana N.Brás, Eduardo J. S.Myllymaa, SamiBrouzgou, ΑngelikiNagano, HanatsuBucur, BogdanNashimoto, YujiBudidha, KarthikNeriya, YutaroBuscaglia, MarcoNikoleli, Dimitrios P.Butt, M. AliNishikimi, ToshioBuzuk, MarijoNogueira Rebelo, Rita DanielaCai, JinguangNozariasbmarz, AminCai, XinxiaNunna, Bharath BabuCalabria, DonatoO’Neil, GlenCampos, RuiOjeda, EdilbertoCampuzano, SusanaOld, JulieCapuano, RosamariaOliveira, Hugo M.Carminati, MarcoOlthuis, WouterCaro, CarlosOrgaz, Eva VargasCarreras, JoaquimOsma, JohannCasals Terre, JasminaOstrovidov, SergeCataldi, PietroPachauri, VivekCavaleiro Rufo, JoãoPadrão, JorgeCernat, AndreeaPala, NezihChang, Chia-ChenPalanisamy, SelvakumarChang, DingranPan, HouwenChen, Jian-LianPan, MatthewChen, LingxinPan, TaisongChen, Shiuan-YehPardy, TamasChianella, IvaParedes-Madrid, LeonelChinnadayyala, Somasekhar R.Park, Jae-YeongChochlakis, DimosthenisPark, Ki SooChoi, Jane RuPark, MinChoi, JungilParupudi, TejasviChoo, JaebumPauk, JolantaChristensen, Jørn BPeckham, Gabriel D.Chung, Meng TingPedrero, MariaChymkowitch, PierrePei, WeihuaCiampi, SimonePemberton, RoyCincotto, Fernando HenriquePeón, JuanjoClement, YuenPerdigão, JoãoColicino, ElenaPeriwal, PriyankaConsidine, MichaelPerreault, JonathanCoppola, SaraPeter, Van Der WalCortese, BarbaraPetrescu, CatalinCosta-Rama, EstefaníaPetti, LuisaCostello, Ben De LacyPiekarz, IlonaCragg, PeterPierpaoli, MattiaCrespi, StefanoPinelli, MicheleCruickshank-Quinn, CharmionPing, JianfengCui, XingranPingarrón, JoséCulshaw, BrianPiro, BenoîtD’Agata, RobertaPirola, LucianoD’Emilia, GiulioPleşca, AdrianD’Sa, RaechellePleshakova, Tatyana O.D’Souza, Rochelle C. J.Plikusiene, IevaDaems, DevinPolo, FedericoDai, JingPoltorak, LukaszDamiati, SamarPoltronieri, PalmiroDaniele, SalvatorePostnikov, Pavel S.David, Iulia GabrielaPrabhakar, AmlenduDavis, FrankPratsinis, Sotiris E.De La Rica, RobertoPrieto-Simon, BeatrizDe Oliveira, Helinando PequenoPrimiceri, ElisabettaDe Souza Gil, EricPriye, AashishDebRoy, ChitritaProchazka, MarekDe-los-Santos-Álvarez, NoemíPucek, AgataDempsey, EithnePushpavanam, KarthikDér, AndrásQiang, YuhaoDhanjai, DhanjaiQin, KairongDi Liegro, ItaliaQuang Trung, TranDi Masi, SabrinaRackauskas, SimasDi Nardo, FabioRamanavicius, ArunasDíaz, StevenRaudabaugh, DanielDickert, Franz L.Raymundo-Pereira, Paulo A.Dilonardo, ElenaReal-Fernández, FelicianaDina, Nicoleta ElenaReddy, M.V.Ding, YichunReyes Vera, ErickDing, ZhaoyangRhee, Jong IlDinh, ToanRho, Hoon SukDodani, Sheel CRiccardi, ClaudiaDoeven, EganRichtera, LukášDominguez, Rocio B.Rinaldi, RosariaDossi, CarloRobert, DanielDragonieri, SilvanoRobertson, Joseph W. F.Drese, Klaus StefanRocha, PauloDu, XinRodríguez-Delgado, Melissa M.Duhamel, JeanRoncada, PaolaDumitru, LiviuRosati, GiulioDymond, MarcusRotariu, LucianDzantiev, B. B.Rothbauer, MarioDzolic, ZoranRoy, DhruvajyotiEady, MatthewRozwadowski, TomaszElena, HusanuRudnicki, KonradElida Nora, FerriRudnitskaya, AlisaEl-Moghazy, AhmedRuiz-Valdepeñas Montiel, VíctorEscobedo, CarlosSaba, SumbalEsposito, FlavioSakač, NikolaEvani, Shankar JaikishanSakkalis, VangelisFahrentrap, JohannesSaltas, VassiliosFan, YuxiaSamaras, IoannisFarka, ZdeněkSantiago, IbonFebbraio, FerdinandoSantos, AdrianoFenton, BrockSantos, KlebsonFernandez, CarlosSarry, FrédéricFerreiro-González, MartaSatoh, HisashiFerrero Martín, Francisco J.Savagatrup, SucholFidan, IsmailScalona, EmiliaFilip, JaroslavScaramuzza, MatteoFlores Johnson, Emmanuel AlejandroSchintke, SilviaFocsan, MonicaSchirhagl, RomanaFologea, DanielSchneider, RudolfFruhbeck, GemaSegecova, AnnaGadeau, Alain-PierreSemancik, StephenGalan, Ana-MariaSensi, MatteoGamella Carballo, MariaSerafín, VeronicaGan, SamuelSerrano-Pertierra, EstherGarcía, TaniaSesen, MuhsincanGartia, Manas RanjanSESSA, FRANCESCOGebicki, JacekSharafeldin, MohamedGhosal, SambuddhaSharma, AnkurGibbs, DanielSharma, AshutoshGirolamo, Annalisa DeSharma, Shiv K.Glowacz, AdamShen, DazhongGok, OzgulShi, QiongfengGonkowski, SlawomirShiddiky, MuhammadGorodkiewicz, EwaShumyantseva, Victoria V.Goud, K YugenderSidorenko, AlexanderGrabowska, IwonaSignore, GiovanniGrattieri, MatteoSikora, AdamGrippo, ValentinaSimion, Cristian E.Gualandi, IsaccoSimon, AnnieGuascito, Maria RacheleSina, AbuGui, RijunSkládal, PetrGumuscu, BurcuSlouka, ChristophGuo, JinhongSmulko, JanuszGuo, ShuangzhuangSoloviev, MikhailGuo, ZhiyongSors, Thomas G.Gupta, SanjuSotnikov, Dmitriy V.Gurban, Ana-MariaSoto, PaulGurov, IgorSozhamannan, ShanmugaGuselnikova, Olga AndreevnaSriram, RashmiH. P. M. Santos, JoãoStan, MirunaHainberger, RainerStasiewicz, KarolHan, Guo-ChengStine, Keith J.Han, XueStobart, JillianHanks, TimothyStorm, Fabio AlexanderHarpaz, DorinStrehle, SteffenHashimoto, MichinaoStrobbia, PietroHavran, LudekSu, Wen-HaoHays, John P.Suciu, Bogdan AndreiHeers, HendrikSukeri, AnandhakumarHelmer, DorotheaSun, DuanpingHeo, JinseokSward, KatherineHirasawa, NoriyasuŚwietlik, RomanHirtz, MichaelSys, MilanHong, Ka L.Sysoev, VictorHorvai, GeorgeSzczerska, Malgorzata JędrzejewskaHosu, OanaSzymborski, TomaszHou, YanxiaTabish, TanveerHsieh, KuangwenTaboada Antelo, PabloHsieh, Tung-HsunTabor, ChristopherHsu, Ching-ChiTagad, Harichandra D.Hsu, Huan-HsuanTakamura, YuzuruHu, LaiguiTakemura, KenshinHu, NingTanaka, SatoshiHuang, Genin GaryTang, HongHuang, JingfengTang, JingjingHuang, Tzu-YenTang, LongtengHuang, XiaolinTaylor, SteveHuh, Yun SukTeixeira, Marcos F.S.Iammarino, MarcoTeixeira, Sofia RodriguesIitani, KentaTeodorescu, FlorinaIkeno, ShinyaTertiş, MihaelaIndic, PremanandaThielemann, ChristianeInoue, JunThomas, RogerIqbal, Hafiz M. N.Thompson, MichaelIqbal, Hafiz. M. NTiggelaar, Roald M.Ivanov, Ilia N.Tilocca, BrunoIvanova, Alla V.Timko, Brian P.Iwamoto, MitsumasaTombelli, SaraJaffrezic-Renault, NicoleTonacci, AlessandroJakieła, SławomirTonello, SarahJanegitz, Bruno CamposTsai, Wan-YuJarocka-Cyrta, ElzbietaTsakova-Stancheva, VesselaJasielec, Jerzy J.Tseng, Tsun-MingJeerapan, ItthiponTsytsarev, VassiliyJia, ZheTurner, ClaireJiang, JiahuanUgo, PaoloJiang, XiantaUrusov, Alexander E.João Ramalhosa, MariaValente, Renata Kelly MendesJosé Laguna Sanz, AlejandroValero, EdelmiraJoven, JorgeVanegas, DianaJu, Wei-JhongVassilakou, ToniaJung, Jae HwanVenkatasubramanian, AnandramJung, YongwonVernick, SefiKallioniemi, ElisaVerwilst, PeterKalogianni, DespinaVettoretti, MartinaKang, TaejoonVidic, JasminaKanjilal, BaishaliVilian, A.T.EzhilKarkanis, StavrosVillarreal-Gómez, Luis JesúsKarpoormath, RajshekharVoinea, Gheorghe-DanielKatona, EvaWada, Ken-IchiKauppinen, AnuWall Medrano, AbrahamKawakami, JunWan, ShibiaoKehl, FlorianWang, CemingKholmukhamedov, AndalebWang, JianlongKhoshmanesh, KhashayarWang, LeiKim, Choong-unWang, Mu-chunKim, Sung-HoonWang, SaiKim, SungjunWang, XuefengKim, Tae-HyungWang, YanyanKirken, Robert A.Wang, ZhenxinKirsanov, DmitryWasilewski, TomaszKitahama, YasutakaWatkins, A. NealKnopf, GeorgeWei, GangKokkinos, ChristosWei, LeiKokkinos, VasileiosWei, YanliKolanowski, Jacek L.Wei, ZhenboKopparthy, Varun LingaiahWelearegay, Tesfalem GeremariamKopra, KariWen, ChangbaoKorecka, LucieWen, YangpingKose, UtkuWhyte, GraemeKouchaki, SamanehWilliams, RyanKovács, ZoltánWilson, Alphus DKowalska, Aneta AnielaWojnowski, WojciechKrejcar, OndrejWong, Danny K. Y.Królicka, AgnieszkaWu, Ching-ChouKulkarni, AtulWu, ChunshengKulkarni, TanmayWu, JianKumar K B, VinayaWu, JieKumar, AbhishekWu, Shu-PaoKumar, SamirWu, YunlongKumar, ShailabhWunsch, Benjamin H.Kung, Yu-ChunXie, KefengKusters, IljaXing, ChenKutikhin, Anton G.Xu, LishengKwon, Seong JungXu, YiLa Spada, LuigiXue, TianyuLachman-Senesh, NoaYan, ShengLaganà, PasqualinaYang, ChengLagugne-Labarthet, FrancoisYang, Chih-TsungLai, MeimeiYang, HaifengLang, JochenYang, Hsiao-YuLang, TingtingYang, Po-ChihLebègue, EstelleYang, YichaoLee, DonghyunYashchenok, AlexeyLee, JunseopYe, HaihangLee, Seok JaeYehl, KevinLeonardi, FrancescaYi, ZaoLeon-Velarde, Carlos GYo, TanakaLeung, KaHoYoon, HyungchulLevine, MindyYoon, JinhoLi, BinxingYoshihara, ToshitadaLi, FengYu, FabiaoLi, JieYu, JingxianLi, ShuangmingYuan, ZhongyunLi, ShunboZacconi, FlaviaLi, WanwanZakaria, A. B. M.Li, ZhenqingZanchetta, GiulianoLiao, XinqinZangheri, MartinaLin, MingyueZarejousheghani, MashaalahLin, Tzu-EnZastrow, Melissa L.Lin, YiliangZeng, MinxiangLinacero, RosarioZhang, DimingLindsay, RobbinZhang, JintaoLisak, GrzegorzZhang, JinyangLiu, Chao-LinZhang, JiweiLiu, GuozhenZhang, LongLiu, JuewenZhang, XianLiu, MeilingZhang, XiuliLiu, MinminZhang, Ye-WangLiu, YuZhang, ZhenLiu, YuelingZhao, HuiLo Faro, Maria JosèZheng, GuangchaoLobo, AndersonZheng, XintingLomonaco, TommasoZherdev, Anatoly V.Lopez Martinez, Maria JoséZhou, HaiboLópez-Pastor, Jose-AntonioZhou, JiajingLosada-Perez, PatriciaZhou, JianLu, XianboZhou, XiaohongLu, XiaoquanZhu, Xiao-SongLucas, Guilherme B.Zhu, ZhiguangLuchian, TudorZhuangqiang, GaoLunelli, LorenzoZieliński, JacekLuo, YangZikria, Yousaf BinMa, GuojiaZiółkowski, RobertMa, HanbinZiyatdinova, GuzelMaccaferri, Nicolò


